# Upper Instrumented Vertebra Selection Influences Proximal Balance but Not Long-Term Clinical Outcomes in Lenke Type 1 Adolescent Idiopathic Scoliosis

**DOI:** 10.3390/jcm15062092

**Published:** 2026-03-10

**Authors:** Evren Karaali, Osman Çiloğlu, Oğuzhan Çiçek, Burak Keklikçioğlu, Hakan Uslu, Mesut Uluöz, Abdülselam Tarhan

**Affiliations:** Department of Orthopedics and Traumatology, Adana City Training and Research Hospital, Adana 01140, Turkey; osmanciloglu@gmail.com (O.Ç.); oguzhan.cicek21@hotmail.com (O.Ç.); drburakkeklikcioglu@gmail.com (B.K.); hakanusl77@gmail.com (H.U.); mesutuluoz@hotmail.com (M.U.); aselamtarhan47@gmail.com (A.T.)

**Keywords:** adolescent idiopathic scoliosis, Lenke type 1, upper instrumented vertebra, patient-reported outcomes

## Abstract

**Background/Objectives:** Selection of the upper instrumented vertebra (UIV) in Lenke type 1 adolescent idiopathic scoliosis (AIS) influences postoperative proximal balance; however, the long-term clinical relevance of radiographic differences remains uncertain. This study evaluated long-term radiological and clinical outcomes in a homogeneous Lenke type 1 AIS cohort undergoing posterior spinal fusion with UIV selection at T2 or T4. **Methods:** During the study period, 120 consecutive Lenke type 1 AIS patients underwent posterior spinal fusion. Twelve patients who developed major postoperative complications were excluded, resulting in a final cohort of 108 patients (T2: *n* = 61; T4: *n* = 47). Patients requiring posterior column osteotomies were excluded to maintain surgical homogeneity. Radiological parameters, including coronal and sagittal alignment, proximal balance measures, and curve flexibility, were assessed preoperatively, postoperatively, and at a minimum follow-up of five years. Clinical outcomes were evaluated using validated Turkish versions of the Pediatric Quality of Life Inventory (PedsQL) and the Scoliosis Research Society—22 revised questionnaire (SRS-22r). Longitudinal within-group changes were analyzed. **Results:** Substantial correction of the main thoracic curve was achieved postoperatively and maintained at long-term follow-up within each cohort (*p* < 0.001). Preoperative bending radiographs demonstrated preserved curve flexibility, indicating that postoperative alignment differences were not attributable to baseline rigidity. Proximal coronal and shoulder balance parameters improved over time within each cohort, with residual differences observed according to UIV selection. Psychosocial domains of the PedsQL and SRS-22r improved significantly over time within each cohort. In contrast, PedsQL physical functioning scores declined significantly at long-term follow-up (*p* < 0.001), consistent with fusion-related stiffness rather than global clinical deterioration. No clinically meaningful divergence in patient-reported outcome trajectories was observed according to the UIV strategy. **Conclusions:** In Lenke type 1 AIS, UIV selection influences long-term proximal balance-related radiological parameters but does not confer a clinically meaningful advantage in patient-reported outcomes. Both T2 and T4 strategies provide durable deformity correction and sustained multidimensional recovery when guided by individualized criteria. Prospective multicenter investigations are warranted to further clarify the clinical relevance of proximal balance metrics.

## 1. Introduction

Adolescent idiopathic scoliosis (AIS) is the most common form of structural spinal deformity in adolescents, with surgical treatment indicated in patients with progressive curves or significant deformity. Posterior spinal fusion remains the standard surgical approach for thoracic AIS, aiming to achieve durable coronal and sagittal correction while preserving spinal balance and optimizing patient-reported outcomes. Among surgically treated patients, those with Lenke type 1 (main thoracic) curves represent a relatively homogeneous subgroup, yet important variability exists in surgical strategy, particularly with respect to fusion level selection [1,2].

Selection of the upper instrumented vertebra (UIV) in Lenke type 1 AIS has long been recognized as a critical determinant of postoperative shoulder and proximal coronal balance. Previous studies have emphasized the importance of radiographic parameters such as T1 tilt, trunk shift, clavicular or shoulder height asymmetry, and upper thoracic vertebral orientation when determining whether fusion should be extended proximally [3,4,5,6,7]. While extending the fusion to higher thoracic levels (e.g., T2 or T3) may improve proximal balance in selected patients, it also increases fusion length and may theoretically influence postoperative function and perceived stiffness. Conversely, limiting the fusion to lower thoracic levels (e.g., T4) allows for shorter constructs but may carry a risk of residual or postoperative shoulder imbalance [5,8,9].

Despite growing interest in radiographic predictors of postoperative balance, the clinical relevance of residual proximal imbalance remains controversial. Several recent investigations have suggested that radiographic measures of shoulder or coronal imbalance do not consistently correlate with patient-reported outcomes following AIS surgery [10,11,12,13,14]. Patient-reported outcome measures such as the Scoliosis Research Society—22 revised questionnaire (SRS-22r) and the Pediatric Quality of Life Inventory (PedsQL) capture broader dimensions of recovery, including pain, self-image, mental health, and overall quality of life, which may not directly mirror radiographic alignment alone [13,14,15,16]. Consequently, the extent to which differences in UIV selection translate into meaningful differences in long-term clinical outcomes remains unclear.

Importantly, most previous studies evaluating UIV selection have focused either on radiographic endpoints or on short- to mid-term follow-up, and many have included heterogeneous curve types or mixed fusion strategies. There remains a lack of long-term data specifically addressing whether radiological differences associated with UIV selection in Lenke type 1 AIS persist over time and, more importantly, whether such differences have a sustained impact on patient-reported clinical outcomes. This represents a relevant knowledge gap, particularly given the emphasis on individualized surgical decision-making and long-term functional recovery in contemporary AIS management.

Therefore, the purpose of the present study was to evaluate radiological and clinical outcomes in a homogeneous cohort of Lenke type 1 AIS patients undergoing posterior spinal fusion with the upper instrumented vertebra selected at either T2 or T4, with a minimum follow-up of five years. We hypothesized that although UIV selection would be associated with differences in proximal balance-related radiological parameters, these differences would not translate into clinically meaningful differences in patient-reported outcomes. Specifically, we aimed to test the hypothesis that radiological variations in parameters such as T1 tilt, shoulder imbalance, and trunk shift may be observed between fusion strategies, while overall clinical recovery patterns remain comparable.

## 2. Methods

### 2.1. Study Design and Patient Selection

This retrospective cohort study included patients with AIS who underwent posterior spinal fusion at a single tertiary spine center between January 2015 and December 2020. Only patients with Lenke type 1 (main thoracic) curves were included to ensure a homogeneous study population. During the study period, a total of 120 consecutive patients with Lenke type 1 AIS underwent posterior spinal fusion (T2: *n* = 68; T4: *n* = 52). Twelve patients (T2: *n* = 7; T4: *n* = 5) who developed major postoperative complications were excluded from the final radiological and clinical outcome analyses. Patients who developed major postoperative complications were excluded from the longitudinal radiological and patient-reported outcome analyses in order to preserve surgical and recovery homogeneity. Major complications requiring revision surgery may substantially alter long-term recovery trajectories and quality-of-life patterns, independent of UIV selection. Complication rates were comparable between groups (T2: 7/68 vs. T4: 5/52; Fisher’s exact test, *p* = 1.00). The remaining 108 patients constituted the final study cohort (T2: *n* = 61; T4: *n* = 47). Surgical complications observed in the initially treated cohort are summarized in Supplementary Table S1. Patients with non-idiopathic scoliosis, additional structural curves requiring fusion, prior spinal surgery, or incomplete radiographic or clinical follow-up were excluded. Patients requiring additional posterior column osteotomies (Ponte osteotomies) were excluded to maintain surgical homogeneity, as these procedures may influence correction magnitude, postoperative pain, recovery trajectory, and complication profile. All included patients had a minimum follow-up of 5 years. Demographic, radiological, and clinical data were extracted from institutional medical records and imaging archives. The study protocol was approved by the local institutional ethics committee, and informed consent was waived due to the retrospective study design and the use of anonymized patient data.

### 2.2. Radiological Evaluation

Standing full-length posteroanterior and lateral spine radiographs were obtained preoperatively, postoperatively, and at the final follow-up. Coronal curve magnitude, thoracic kyphosis, lumbar lordosis, trunk shift, shoulder imbalance, and T1 tilt angle were measured using standard radiographic techniques. Proximal balance was primarily assessed using trunk shift, shoulder imbalance, and T1 tilt angle, as these parameters are commonly used to evaluate coronal and shoulder balance in thoracic scoliosis and are closely related to UIV selection. All radiographic measurements were performed by a single experienced observer using a digital PACS system (Sectra AB, Linköping, Sweden) standardized measurement protocols. Each parameter was measured twice at separate time points, and mean values were used for analysis.

### 2.3. Assessment of Curve Flexibility

Preoperative curve flexibility was assessed using standing posteroanterior radiographs and preoperative bending radiographs, in accordance with standard practice in AIS surgery. Bending radiographs were obtained to evaluate the reducibility of the main thoracic curve and to support surgical planning. In addition, intraoperative assessment of curve flexibility was performed under general anesthesia using fluoroscopic imaging, allowing evaluation of spontaneous curve correction and segmental alignment in a relaxed state.

Information derived from preoperative and intraoperative flexibility assessment was used to support the determination of the extent of instrumentation, including the selection of fusion levels, with particular attention to overall coronal balance and segmental alignment. These assessments were integrated with established radiographic parameters and surgeon judgment rather than applied as isolated decision criteria.

### 2.4. Upper Instrumented Vertebra Selection

Selection of the UIV was based on preoperative radiographic characteristics and proximal balance considerations, in accordance with commonly accepted principles for Lenke type 1 scoliosis. Fusion was extended to T2 in patients demonstrating preoperative features associated with an increased risk of postoperative proximal imbalance, including clinically evident shoulder asymmetry in conjunction with increased T1 tilt and coronal trunk shift. In contrast, T4 was selected as the UIV in patients with relatively balanced shoulders, lower preoperative T1 tilt, and satisfactory proximal coronal alignment, allowing for a shorter fusion construct while preserving overall balance. UIV selection was individualized for each patient and reflected the surgeon’s judgment aimed at optimizing postoperative proximal balance rather than random assignment. Consequently, differences between the T2 and T4 groups were considered inherent to the clinical decision-making process.

### 2.5. Surgical Technique

All patients underwent posterior spinal fusion using a standard midline posterior approach. Segmental pedicle screw instrumentation (Tasarımmed Medical Devices, Istanbul, Turkey) was applied across the fused levels. Deformity correction was achieved using rod derotation, translation techniques, and selective compression and distraction to restore coronal alignment and thoracic kyphosis. Care was taken to avoid excessive proximal overcorrection in order to maintain shoulder and coronal balance. Local autograft bone obtained during posterior element decortication was used for fusion. No anterior procedures or osteotomies beyond standard posterior releases were performed in this cohort.

All constructs utilized 6.5 mm titanium rods. Pedicle screws of 5.5 mm and 6.5 mm in diameter were used according to patient age and pedicle anatomy at thoracic and lumbar levels, without systematic differences between cohorts. Screw density strategy and correction maneuvers were applied consistently across groups, and the only intended structural difference between cohorts was the selected UIV level. Particular attention was paid to proximal rod contouring and controlled correction maneuvers in order to avoid excessive proximal overcorrection. Intraoperative assessment of shoulder balance and coronal alignment was performed following rod insertion and correction maneuvers, with adjustments made as necessary to maintain harmonious proximal alignment. Fusion levels were determined based on preoperative radiographic characteristics, curve flexibility, and overall coronal balance rather than applying a fixed algorithm.

### 2.6. Postoperative Management and Rehabilitation

All patients followed a standardized postoperative care protocol. Early mobilization was initiated on the first postoperative day under supervision. A thoracolumbosacral orthosis (TLSO) brace was routinely prescribed for approximately 45 days following surgery. The brace was intended to provide temporary external support during the early postoperative phase, facilitating patient confidence during mobilization and daily activities rather than serving as a primary stabilizing measure.

Patients were encouraged to resume daily activities gradually, with avoidance of high-impact sports during the early recovery period. Return to non-contact sports was generally permitted after radiographic evidence of progressive fusion, whereas unrestricted activity was allowed following clinical and radiographic confirmation of stable fusion. Postoperative care and rehabilitation guidance were consistent across cohorts.

### 2.7. Clinical Outcome Assessment

Clinical outcomes were assessed preoperatively and at final follow-up using validated Turkish versions of the PedsQL and SRS-22r questionnaires [17,18]. Both instruments have undergone formal linguistic and cultural validation for Turkish-speaking populations. The questionnaires were completed by the patients in the presence of a parent, who provided assistance when clarification of specific items was required. PedsQL domain scores were calculated according to standard scoring guidelines, with higher scores indicating better health-related quality of life. SRS-22r domain scores were calculated as the mean of the corresponding item responses. The Satisfaction domain of the SRS-22r was not included in longitudinal analyses because it is assessed at a single postoperative time point and therefore is not appropriate for preoperative–postoperative comparison.

### 2.8. Statistical Analysis

Statistical analyses were performed to evaluate changes over time within each cohort and to compare outcomes between UIV strategies. Continuous variables are presented as mean ± standard deviation, and categorical variables are presented as counts and percentages. Longitudinal within-group changes from the preoperative period to the final follow-up were analyzed using paired statistical tests, as appropriate. Formal between-group comparisons of final follow-up values and change-from-baseline (Δ) scores were performed using independent-sample *t*-tests. Effect sizes were calculated using Cohen’s d to quantify the magnitude of between-group differences. A two-sided *p* value < 0.05 was considered statistically significant. All statistical analyses were conducted using SPSS Statistics (version 26.0, IBM Corp., Armonk, NY, USA).

## 3. Results

### 3.1. Patient Characteristics

A total of 108 patients with Lenke type 1 AIS were included in the study, of whom 61 underwent posterior spinal fusion with the UIV at T2 and 47 at T4. Patient characteristics were evaluated descriptively within each cohort. Age at surgery, skeletal maturity as assessed by Risser grade, and duration of follow-up were similar across cohorts. Female patients constituted the majority in each cohort. Mean follow-up exceeded five years in both cohorts. Surgical outcomes and complications were assessed within each group. The incidence of major postoperative complications did not differ between cohorts (T2: 7/68 [10.3%] vs. T4: 5/52 [9.6%]; Fisher’s exact test, *p* = 1.00) (Supplementary Table S2). No cases of pseudarthrosis were observed during the follow-up. Screw misplacement, proximal junctional kyphosis, and the adding-on phenomenon occurred infrequently and were descriptively reported for each cohort (Table 1).

### 3.2. Radiological Outcomes

**Figure 1:**
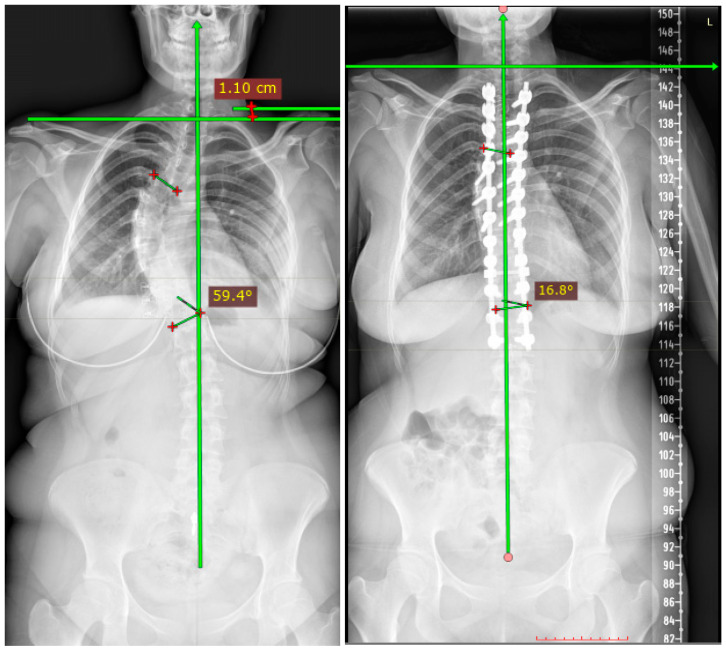
Representative preoperative and postoperative standing posteroanterior radiographs demonstrating coronal curve correction and proximal balance in a patient fused to T2 as the upper instrumented vertebra. Changes are shown longitudinally within the same patient. “L” indicates the left side of the patient, and “+” marks the reference points used for angular measurement.

**Figure 2:**
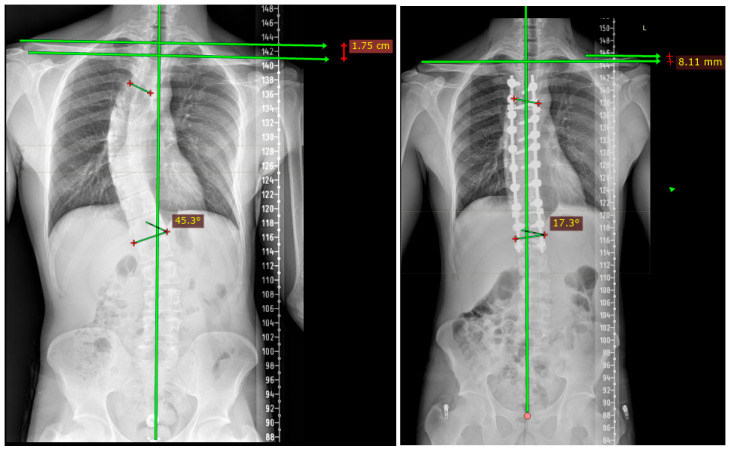
Representative preoperative and postoperative standing posteroanterior radiographs demonstrating coronal curve correction and proximal balance in a patient fused to T4 as the upper instrumented vertebra. Changes are shown longitudinally within the same patient. “L” indicates the left side of the patient, and “+” marks the reference points used for angular measurement.

Radiological outcomes were evaluated longitudinally within each cohort (Table 2). In each fusion group, substantial correction of the main thoracic curve was achieved postoperatively and maintained at final follow-up. Within-group analyses demonstrated statistically significant reductions in standing coronal Cobb angles from the preoperative period to final follow-up in each cohort (*p* < 0.001). Preoperative bending radiographs demonstrated marked curve flexibility within each cohort. The flexibility index indicated that preoperative curve flexibility was preserved across patients, supporting that postoperative alignment changes were not driven by baseline rigidity (Table 2). Representative preoperative and postoperative radiographs illustrating coronal correction and proximal balance in patients fused to T2 and T4 are shown in Figure 1 and Figure 2, respectively.

Sagittal profile parameters were similarly assessed over time within each group. Thoracic kyphosis increased significantly after surgery and remained stable at final follow-up in each cohort (*p* < 0.001). Lumbar lordosis demonstrated small but statistically significant increases from the preoperative period to final follow-up within each cohort, without evidence of progressive sagittal malalignment. Parameters related to proximal coronal and shoulder balance showed time-dependent improvement within each cohort. Reductions in trunk shift, shoulder imbalance, and T1 tilt were observed postoperatively and maintained at final follow-up. The magnitude of residual proximal balance parameters varied according to the selected UIV and is presented descriptively. Categorical assessment of sagittal alignment at final follow-up demonstrated that the majority of patients in each cohort remained sagittally neutral. Positive or negative sagittal imbalance was uncommon and observed only in a small number of patients, without a consistent pattern over time (Supplementary Table S3).

### 3.3. Clinical Outcomes

Clinical outcomes were analyzed longitudinally within each cohort. From the preoperative period to the final follow-up, statistically significant improvements were observed over time in multiple psychosocial domains of the PedsQL, including emotional, social, and school functioning. Similarly, SRS-22r domains related to pain, self-image, and mental health demonstrated significant improvement within each cohort. In contrast, PedsQL physical functioning scores demonstrated a statistically significant decline over time within each cohort (*p* < 0.001). This pattern reflects changes in physical functioning following spinal fusion, rather than a deterioration in overall clinical status. Despite variability in radiological parameters related to proximal balance, overall trajectories of patient-reported outcome measures over time were similar in direction across cohorts. No clinically meaningful divergence in health-related quality-of-life recovery patterns was identified when outcomes were assessed longitudinally within each cohort (Table 3).

Formal between-group comparisons of final values demonstrated significantly lower trunk shift, shoulder imbalance, and T1 tilt in the T2 cohort at the last follow-up. However, no statistically significant between-group differences were observed in PedsQL domains or most SRS-22r domains, with only small effect sizes noted in isolated parameters. Comparison of change-from-baseline (Δ) values revealed similar improvement trajectories in patient-reported outcomes between cohorts. Detailed between-group statistical analyses are presented in Supplementary Table S4.

Formal between-group comparisons of final values and change-from-baseline (Δ) scores are presented in Supplementary Table S4.

## 4. Discussion

The present study evaluated the long-term radiological and clinical outcomes of posterior spinal fusion in a homogeneous cohort of Lenke type 1 AIS patients, with particular emphasis on UIV selection at T2 versus T4. The principal finding is that, although UIV selection was associated with expected differences in proximal balance-related radiological parameters, these differences did not translate into clinically meaningful differences in patient-reported outcomes at a minimum follow-up of five years.

Consistent with prior reports, substantial and durable coronal correction was achieved postoperatively and maintained over time within each cohort, with preservation of global sagittal alignment [1,2,8]. Restoration and maintenance of thoracic kyphosis and lumbar lordosis indicate that neither proximal fusion strategy adversely affected sagittal balance in Lenke type 1 curves [2,5]. These observations support the concept that contemporary posterior correction techniques provide reliable long-term radiographic stability across different fusion-level strategies when applied using established principles [8,9].

As anticipated, parameters related to proximal coronal and shoulder balance demonstrated time-dependent improvement within each cohort, with the magnitude of residual trunk shift, shoulder imbalance, and T1 tilt varying according to the selected UIV. These findings align with prior work emphasizing the relevance of proximal balance parameters in guiding fusion-level selection in thoracic AIS [3,4,5,6,7]. Extending fusion proximally has been advocated in patients with preoperative features associated with an increased risk of postoperative shoulder imbalance, such as elevated shoulders, increased T1 tilt, or significant coronal trunk deviation [5,6]. The present data demonstrate that differences in proximal balance metrics persist at long-term follow-up, reinforcing the durable radiographic impact of the UIV strategy.

Preoperative bending radiographs demonstrated substantial flexibility of the main thoracic curve, and the calculated flexibility index indicated preserved reducibility within each cohort. This observation is clinically relevant, as curve flexibility is routinely integrated into fusion-level planning in Lenke type 1 AIS to optimize balance while avoiding unnecessary extension of instrumentation [1,2]. In this context, the observed long-term differences in proximal balance parameters are more plausibly attributable to UIV selection and proximal alignment considerations rather than baseline curve rigidity.

Despite variability in proximal balance-related radiological parameters, longitudinal patterns of clinical recovery were favorable within each cohort. Improvements over time were observed in multiple domains of health-related quality of life, pain, self-image, and mental health, as assessed by the PedsQL and SRS-22r instruments. Prior studies have similarly reported a dissociation between radiographic alignment and patient-reported outcomes, suggesting that objective measures of balance do not consistently predict patient-perceived surgical success [11,12,13,14].

This dissociation underscores the multifactorial nature of postoperative recovery in AIS. While radiographic parameters provide an objective assessment of deformity correction and alignment, patient-reported outcomes reflect broader physical, psychological, and social dimensions of health [13,14,15]. In this setting, modest residual differences in proximal balance may be well tolerated and may not meaningfully influence long-term quality of life. The present findings add long-term evidence supporting this concept in a strictly defined Lenke type 1 population [15,16].

An additional observation concerns the trajectory of physical functioning. Although overall recovery patterns were favorable, physical functioning scores demonstrated a decline over time within each cohort. This domain-specific pattern is consistent with reduced spinal mobility after fusion, long-term activity modification, and adaptive movement behaviors, rather than inadequate deformity correction or surgical failure [16]. Importantly, between-group comparisons demonstrated similar change-from-baseline trajectories and small effect sizes, suggesting that this decline does not represent a strategy-specific disadvantage. Furthermore, previously reported minimal clinically important difference (MCID) thresholds for pediatric quality-of-life instruments indicate that numerical score changes do not necessarily equate to clinically meaningful deterioration. This decline occurred alongside significant improvements in psychosocial and pain-related domains, suggesting that long-term recovery in AIS reflects adaptation and recalibration of physical expectations rather than a global deterioration in health status. The standardized use of a temporary TLSO brace during the early postoperative period may also have contributed to adaptive movement behaviors and activity modification in the short term. However, given the relatively brief duration of brace use, the observed long-term trajectory in physical functioning is more likely attributable to fusion-related stiffness and biomechanical adaptation rather than external immobilization alone.

Beyond quantitative radiographic metrics, assessment of shoulder balance itself is heterogeneous and depends on the measurement technique and observer perspective. Recent studies have shown that cosmetic shoulder balance may vary when evaluated from different viewpoints, highlighting that radiographic symmetry does not always capture patient-perceived appearance or satisfaction [19]. This consideration is particularly relevant for UIV selection, where surgical planning often targets measurable indices such as T1 tilt or shoulder height difference, yet patient-facing outcomes may be influenced more by global appearance and psychosocial adaptation than by a single radiographic parameter.

Interpretation of patient-reported outcomes should likewise consider instrument-specific characteristics and limitations. Although the SRS-22r is widely used and endorsed for AIS, it may not fully capture nuanced cosmetic concerns or activity-specific limitations, particularly in long-term follow-up [17]. Similarly, while the PedsQL provides a validated and culturally adapted assessment of general health-related quality of life in adolescents [18], it is not deformity-specific and may be less sensitive to subtle changes in radiographic balance parameters. These considerations offer a plausible explanation for the present findings: UIV selection can modify proximal balance metrics in a measurable manner, whereas the downstream effect on patient-reported recovery may be moderated by patient adaptation and by the scope and sensitivity of the instruments employed.

From a broader perspective, postoperative shoulder balance has long been described as an “elusive” endpoint in AIS surgery, reflecting the complex interaction among curve pattern, correction magnitude, proximal thoracic alignment, and fusion-level selection [20,21]. Prior investigations examining fusion to T2, T3, or T4 have reported radiographic differences related to shoulder balance, yet consistent clinical superiority of one level over another has been difficult to demonstrate across heterogeneous cohorts [22]. By focusing on a homogeneous Lenke type 1 population with long-term follow-up, the present study supports contemporary guidance favoring individualized fusion-level selection rather than a uniform strategy [23]. This approach is best contextualized within the Lenke classification framework, which remains foundational for understanding curve behavior and tailoring surgical planning in AIS [10,24].

This study has several notable strengths. The analysis was restricted to a homogeneous Lenke type 1 cohort, minimizing confounding related to curve heterogeneity and enabling focused evaluation of the UIV strategy. Surgical technique and postoperative management were standardized, and patients requiring posterior column osteotomies or major revision procedures were excluded to preserve procedural consistency. The minimum follow-up of five years allowed assessment of the durability of radiographic alignment and longitudinal patient-reported outcomes. Finally, integration of objective radiological parameters with validated, culturally adapted outcome measures provides a comprehensive perspective on both structural correction and patient-perceived recovery.

Several limitations warrant consideration. First, the retrospective, single-center design may limit generalizability and introduce inherent selection bias. In addition, UIV selection was not randomized but based on preoperative radiographic characteristics and surgeon judgment. Furthermore, patients requiring posterior column osteotomies or those who developed major postoperative complications were excluded to preserve surgical and recovery homogeneity. Major complications, particularly those requiring revision surgery, may substantially alter long-term recovery trajectories and quality-of-life patterns independent of UIV selection. Although complication distribution was comparable between cohorts, exclusion of these cases may limit generalizability to more complex recovery pathways. In addition, validated outcome measures were used, and therefore, subtle cosmetic perceptions and activity-specific limitations could not be fully captured. Although the sample size was moderate, effect size estimates were generally small across patient-reported outcome domains, suggesting that the absence of statistically significant between-group differences is unlikely to reflect a masked clinically meaningful effect. In addition, radiographic measurements were performed by a single observer. Although each parameter was measured twice and mean values were used for analysis to enhance internal consistency, formal interobserver reliability analysis was not conducted. This represents a methodological limitation.

## 5. Conclusions

In Lenke type 1 AIS, UIV selection influences long-term proximal balance-related radiological parameters. However, formal between-group comparisons of final follow-up values and change-from-baseline trajectories demonstrated no substantial divergence in patient-reported outcomes between T2 and T4 strategies, with generally small effect sizes observed across PRO domains. Both T2 and T4 fusion strategies achieve durable deformity correction and sustained multidimensional recovery when guided by individualized radiographic and clinical criteria. These findings underscore the importance of tailored fusion-level planning while avoiding overinterpretation of isolated balance metrics as determinants of long-term patient-perceived success. Future prospective, multicenter investigations incorporating standardized fusion-level algorithms and extended patient-centered follow-up are warranted to further clarify the clinical relevance of proximal balance parameters in AIS.

## Figures and Tables

**Table 1:** Baseline demographic, surgical, and follow-up characteristics of Lenke type 1 adolescent idiopathic scoliosis patients according to upper instrumented vertebra selection.

Variables	T2 (*n* = 61)	T4 (*n* = 47)
Patient demographics and maturity		
Age at surgery (years)	13.9 ± 1.4	14.2 ± 1.6
Male sex, *n* (%)	20 (32.8)	12 (25.5)
Female sex, *n* (%)	41 (67.2)	35 (74.5)
Risser grade at surgery	3.2 ± 0.9	3.0 ± 0.8
**Follow-up characteristics**		
Follow-up duration (months)	64.1 ± 4.3	63.6 ± 4.2
**Anthropometric parameters**		
Height—preoperative (cm)	159.3 ± 5.8	158.8 ± 6.0
Height—postoperative (cm)	163.9 ± 6.1	162.4 ± 6.3

Data are presented as mean ± standard deviation or *n* (%). All patients had Lenke type 1 (main thoracic) adolescent idiopathic scoliosis. Upper instrumented vertebra selection (T2 vs. T4) was based on preoperative curve characteristics and proximal balance considerations.

**Table 2:** Radiological outcomes related to proximal balance according to upper instrumented vertebra selection in Lenke type 1 AIS.

Parameter	Group	Preoperative	Postoperative	Last Follow-Up	P (Within-Group)
Coronal curve magnitude—standing (°)	T2	52.9 ± 7.3	8.1 ± 4.0	8.4 ± 4.1	<0.001
	T4	52.0 ± 7.0	9.8 ± 6.4	9.9 ± 6.7	<0.001
Coronal curve magnitude—bending (°)	T2	35.5 ± 5.5	—	—	—
	T4	33.3 ± 4.6	—	—	—
Flexibility index (%)	T2	32.9 ± 8.4	—	—	—
	T4	36.0 ± 7.9	—	—	—
Thoracic kyphosis (°)	T2	17.8 ± 3.1	27.6 ± 4.3	26.6 ± 4.0	<0.001
	T4	18.0 ± 3.3	26.9 ± 3.7	27.0 ± 3.9	<0.001
Trunk shift (mm)	T2	28.8 ± 6.5	5.7 ± 2.7	5.4 ± 2.9	<0.001
	T4	29.2 ± 6.7	6.9 ± 3.4	6.8 ± 3.6	<0.001
Shoulder imbalance (mm)	T2	12.1 ± 3.7	2.9 ± 3.3	2.5 ± 3.2	<0.001
	T4	12.4 ± 3.6	4.3 ± 3.3	4.1 ± 3.0	<0.001
T1 tilt angle (°)	T2	8.7 ± 2.6	4.0 ± 1.9	4.3 ± 1.7	<0.001
	T4	6.9 ± 2.4	4.7 ± 1.9	4.9 ± 1.7	<0.001
Lumbar lordosis (°)	T2	46.0 ± 5.2	48.4 ± 4.6	49.4 ± 4.5	0.01
	T4	45.8 ± 5.1	48.0 ± 4.2	48.5 ± 4.0	0.02

**Table 3:** Clinical outcome changes in Lenke type 1 AIS patients after posterior spinal fusion.

Outcome Domain	Group	Preoperative Mean ± SD	Last Follow-Up Mean ± SD	P (Within-Group)
PedsQL—Physical Functioning	T2 (*n* = 61)	94.1 ± 6.7	81.5 ± 7.4	<0.001
	T4 (*n* = 47)	93.3 ± 6.1	79.8 ± 7.9	<0.001
PedsQL—Emotional Functioning	T2	84.4 ± 10.2	94.9 ± 6.1	<0.001
	T4	81.9 ± 11.8	93.8 ± 6.9	<0.001
PedsQL—Social Functioning	T2	95.3 ± 5.1	91.1 ± 6.8	0.002
	T4	93.2 ± 7.2	90.7 ± 7.5	0.004
PedsQL—School Functioning	T2	66.6 ± 11.2	88.6 ± 7.9	<0.001
	T4	66.4 ± 14.1	88.3 ± 8.4	<0.001
SRS-22r—Function	T2	4.15 ± 0.21	4.22 ± 0.19	0.010
	T4	3.98 ± 0.26	4.10 ± 0.22	0.020
SRS-22r—Pain	T2	4.04 ± 0.33	4.85 ± 0.18	<0.001
	T4	3.94 ± 0.36	4.78 ± 0.21	<0.001
SRS-22r—Self-Image	T2	4.31 ± 0.27	4.88 ± 0.15	<0.001
	T4	4.18 ± 0.25	4.87 ± 0.17	<0.001
SRS-22r—Mental Health	T2	3.86 ± 0.32	4.73 ± 0.20	<0.001
	T4	3.71 ± 0.33	4.66 ± 0.23	<0.001

Data are presented as mean ± standard deviation. Clinical outcomes were assessed preoperatively and at last follow-up. Significant within-group clinical improvement was observed over time in each cohort. The Satisfaction domain of the SRS-22r was not analyzed due to its single-time-point nature.

## Data Availability

The data supporting the findings of this study are not publicly available due to ethical restrictions and protection of patient privacy.

## Supplementary Materials

The following supporting information can be downloaded at https://www.mdpi.com/article/10.3390/jcm15062092/s1, Table S1: Surgical complications observed in the initially treated cohort (*n* = 120); Table S2: Distribution of Major Postoperative Complications According to Upper Instrumented Vertebra Selection; Table S3: Sagittal alignment at last follow-up, *n* (%); Table S4: Between-Group Comparisons of Final and Change-from-Baseline Outcomes According to UIV Selection.

## References

[1] Baghdadi S., Baldwin K. (2024). Selection of fusion levels in adolescent idiopathic scoliosis. Curr. Rev. Musculoskelet. Med..

[2] Chen R., Li Y., Wang T., Wang A., Ma Z., Liang M., Yuan S., Zang L., Fan N. (2026). Advances in fusion level selection and surgical approaches for adolescent idiopathic scoliosis based on the Lenke classification system: A narrative review. BMC Surg..

[3] Qdn V., Hhh N., Ht N., Bn P., Tk T. (2024). Shoulder and neck balance in adolescent idiopathic scoliosis: Which radiographic indices are reliable and practical?. Malays. Orthop. J..

[4] Jiang J., Chen X., Qiu Y., Wang B., Yu Y., Zhu Z.-Z. (2022). Postoperative shoulder balance in Lenke type 1 adolescent idiopathic scoliosis patients with large thoracic curves (Cobb ≥ 70°): A radiographic study. BMC Musculoskelet. Disord..

[5] Chan C.Y.W., Hong N.C., Chandirasegaran S., Chiu C.K., Kwan M.K. (2025). The influence of relative curve correction and upper instrumented vertebra tilt angle on postoperative shoulder balance following posterior spinal fusion in Lenke type 1 and 2 adolescent idiopathic scoliosis. Glob. Spine J..

[6] Ramchandran S., Pierce A., Callan C., Ramzanian T., Mohile N., Keshavarzi S., Errico T., George S. (2024). Does levelling of T1 tilt intraoperatively affect postoperative shoulder balance in adolescent idiopathic scoliosis patients?. Spine Deform..

[7] Jiang Z., Wang H., Cui R., Wang X., Wang Y., Sun M., Peng F., Li T., Zhang W., Zhang W. (2024). Correlation analysis and clinical significance of changes in upper thoracic vertebra tilt and clavicle angle pre- and post-operation. Front. Surg..

[8] Pishnamaz M., Migliorini F., Blume C., Kobbe P., Trobisch P., Delbrück H., Hildebrand F., Herren C. (2024). Long-term outcomes of spinal fusion in adolescent idiopathic scoliosis: A literature review. Eur. J. Med. Res..

[9] Park S.J., Park J.S., Kang D.H., Lee C.S. (2024). The optimal lowest instrumented vertebra to prevent the distal adding-on phenomenon in patients undergoing selective thoracic fusion for adolescent idiopathic scoliosis. J. Clin. Med..

[10] van Royen B.J.B. (2023). Understanding the Lenke classification for adolescent idiopathic scoliosis. Curr. Probl. Diagn. Radiol..

[11] Xie F., Bai M., Zhu X., Yang G., Ling J., Li Y., Wang F., Wang Y., Luo Z., Hu X. (2025). How much postoperative shoulder imbalance is satisfactory for Lenke 1–2 adolescent idiopathic scoliosis patients?. Spine Res..

[12] de Barros A.G.C., Almeida G.J., Alves G.F., Leal A.C., Carelli L.E. (2025). Beyond the Cobb angle: Radiographic measurements of clavicular angle, T1 tilt, and trunk misalignment. Eur. Spine J..

[13] Alamrani S., Labelle H., Gardner A., Falla D., Russell E., Rushton A.B., Heneghan N.R. (2021). Content validity of the Scoliosis Research Society questionnaire-22 revised (SRS-22r) for adolescents with idiopathic scoliosis: Protocol for a qualitative study exploring patients’ and practitioners’ perspectives. BMJ Open.

[14] Alamrani S., Gardner A., Falla D., Russell E., Rushton A.B., Heneghan N.R. (2023). Content validity of the Scoliosis Research Society questionnaire (SRS-22r): A qualitative concept elicitation study. PLoS ONE.

[15] Chan K.C.A., Wong S.L., Cheung J.P.Y., Cheung P.W.H. (2025). Relationships of PedsQL 4.0 generic core scales with other validated HRQoL instruments in braced idiopathic scoliosis patients: An age and curve severity-specific analysis. J. Orthop. Surg..

[16] Lindsay S.E., Thompson A., Hummel J., Halsey M.F., Yang S. (2024). Adolescent perception of stiffness after spinal fusion surgery. J. Patient Exp..

[17] Alanay A., Cil A., Berk H., Acaroglu R.E., Yazici M., Akcali O., Kosay C., Genc Y., Surat A. (2005). Reliability and validity of the adapted Turkish version of the Scoliosis Research Society-22 (SRS-22) questionnaire. Spine.

[18] Memik N.C., Ağaoğlu B., Coşkun A., Uneri O.S., Karakaya I. (2007). The validity and reliability of the Turkish Pediatric Quality of Life Inventory for children 13–18 years old. Turk Psikiyatri Derg..

[19] Liang B., Li D., Li J., Hu Z., Xu Y., Liu C., Qiu Y., Zhu Z., Liu Z. (2025). Evaluation of cosmetic shoulder balance in Lenke type 1 and 2 adolescent idiopathic scoliosis across different observational perspectives. Quant. Imaging Med. Surg..

[20] Brooks J.T., Bastrom T., Bartley C., Lonner B.S., Shah S.A., Miyanji F., Asghar J., Newton P.O., Yaszay B. (2018). In search of the ever-elusive postoperative shoulder balance: Is the T2 UIV the key?. Spine Deform..

[21] Drake L.C., D’Amore P.W., Fontenot B., Tetreault T.A., Younis M., Leonardi C., Valenzuela-Moss J., Andras L.M., Heffernan M.J. (2025). Rule breakers achieve successful shoulder balance following posterior spinal fusion for adolescent idiopathic scoliosis. Spine Deform..

[22] Zhao J., Chen Z., Yang M., Li G., Zhao Y., Li M. (2018). Does spinal fusion to T2, T3, or T4 affect sagittal alignment of the cervical spine in Lenke 1 AIS patients: A retrospective study. Medicine.

[23] Trobisch P.D., Ducoffe A.R., Lonner B.S., Errico T.J. (2013). Choosing fusion levels in adolescent idiopathic scoliosis. J. Am. Acad. Orthop. Surg..

[24] Lenke L.G., Betz R.R., Harms J., Bridwell K.H., Clements D.H., Lowe T.G., Blanke K. (2001). Adolescent idiopathic scoliosis: A new classification to determine extent of spinal arthrodesis. J. Bone Jt. Surg. Am..

