# Supplementary material for: Upper Instrumented Vertebra Selection Influences Proximal Balance but Not Long-Term Clinical Outcomes in Lenke Type 1 Adolescent Idiopathic Scoliosis
Source: J Clin Med. 2026 Mar 10;15(6):2092. doi: 10.3390/jcm15062092 (PMC13027300; doi:10.3390/jcm15062092)
Supplement: Supplementary file 1 [file jcm-15-02092-s001.zip › jcm-4170546-supplementary.pdf]

Table S1. Surgical complications observed in the initially treated cohort (*n* = 120).

Variable	T2 (n = 68)	T4 (n = 52)
**Screw misplacement, n (%)**	1 (1.5)	2 (3.8)
**Proximal junctional kyphosis, n (%)**	2 (2.9)	0 (0)
**Pseudarthrosis, n (%)**	2 (2.9)	2 (3.8)
**Adding-on phenomenon, n (%)**	2 (2.9)	1 (1.9)

Table S2 Distribution of Major Postoperative Complications According to Upper Instrumented Vertebra Selection.

Complication Status	T2 (n = 68)	T4 (n = 52)	p-Value
Major complication	7 (10.3%)	5 (9.6%)	1.00*
No major complication	61 (89.7%)	47 (90.4%)	

* Fisher's exact test (two-sided).

Table S3. Sagittal alignment at last follow-up, *n* (%).

Sagittal Alignment (Last FU)	T2 (*n* = 61)	T4 (*n* = 47)
Neutral	58 (95.1)	44 (93.6)
Positive	2 (3.3)	0 (0)
Negative	1 (1.6)	3 (6.4)

Data are presented as mean ± standard deviation or *n* (%). Preoperative bending radiographs were obtained to assess curve flexibility. The flexibility index was calculated as [(standing Cobb − bending Cobb)/standing Cobb] × 100. *p* values reflect within-group comparisons between the preoperative period and final follow-up. Sagittal alignment was evaluated categorically at final follow-up and is presented descriptively.

Table S4. Between-Group Comparisons of Final and Change-from-Baseline Outcomes According to UIV Selection.

Outcome	Final T2 (mean ± SD)	Final T4 (mean ± SD)	p (Final)	Cohen's d (Final)	Δ T2	Δ T4	p (Δ)	Cohen's d (Δ)
**Radiological Outcomes**								
Coronal Cobb (°)	8.4 ± 4.1	9.9 ± 6.7	0.15	0.28	−44.5	−42.1	0.21	0.22
Thoracic kyphosis (°)	26.6 ± 4.0	27.0 ± 3.9	0.62	0.10	+8.8	+9.0	0.74	0.05
Trunk shift (mm)	5.4 ± 2.9	6.8 ± 3.6	0.03	0.43	−23.4	−22.4	0.48	0.18
Shoulder imbalance (mm)	2.5 ± 3.2	4.1 ± 3.0	0.02	0.51	−9.6	−8.3	0.31	0.24
T1 tilt (°)	4.3 ± 1.7	4.9 ± 1.7	0.04	0.35	−4.4	−2.0	0.01	0.60
Lumbar lordosis (°)	49.4 ± 4.5	48.5 ± 4.0	0.29	0.21	+3.4	+2.7	0.41	0.15
**Patient-Reported Outcomes**								
PedsQL Physical	81.5 ± 7.4	79.8 ± 7.9	0.23	0.22	−12.6	−13.5	0.66	0.11
PedsQL Emotional	94.9 ± 6.1	93.8 ± 6.9	0.41	0.17	+10.5	+11.9	0.52	0.14
PedsQL Social	91.1 ± 6.8	90.7 ± 7.5	0.78	0.05	−4.2	−2.5	0.29	0.23
PedsQL School	88.6 ± 7.9	88.3 ± 8.4	0.87	0.04	+22.0	+21.9	0.96	0.01
SRS Function	4.22 ± 0.19	4.10 ± 0.22	0.01	0.59	+0.07	+0.12	0.18	0.24
SRS Pain	4.85 ± 0.18	4.78 ± 0.21	0.04	0.36	+0.81	+0.84	0.72	0.08
SRS Self-Image	4.88 ± 0.15	4.87 ± 0.17	0.79	0.06	+0.57	+0.69	0.11	0.38
SRS Mental Health	4.73 ± 0.20	4.66 ± 0.23	0.09	0.32	+0.87	+0.95	0.28	0.21

Data are presented as mean ± standard deviation. Δ indicates change from baseline (final follow-up minus preoperative value). Between-group comparisons were performed using independent-samples t-tests. Effect sizes were calculated using Cohen's d.
